# Prevalence and anticoccidial drug sensitivity of *Eimeria tenella* isolated from commercial broiler farms in Thailand

**DOI:** 10.14202/vetworld.2025.1561-1570

**Published:** 2025-06-15

**Authors:** Suttitas Tongkamsai, Siraprapa Boobphahom, Rachan Apphaicha, Niwat Chansiripornchai

**Affiliations:** 1 Department of Veterinary Medicine, Faculty of Veterinary Science, Rajamangala University of Technology Tawan-Ok, Chonburi, Thailand; 2 Research Center for Neuroscience, Institute of Molecular Biosciences, Mahidol University, Nakhon Pathom, Thailand; 3 Avian Health Research Unit, Department of Veterinary Medicine, Faculty of Veterinary Science, Chulalongkorn University, Bangkok, Thailand

**Keywords:** anticoccidial sensitivity, broiler chickens, coccidiosis, drug resistance, *Eimeria tenella*, Thailand

## Abstract

**Background and Aim:**

Coccidiosis, a parasitic disease caused by Eimeria species, remains a critical challenge for poultry production worldwide. *Eimeria tenella* is one of the most pathogenic species, causing intestinal lesions and compromising growth in chickens. This study aimed to estimate the prevalence of *Eimeria* species and evaluate the anticoccidial drug sensitivity of *E. tenella* field isolates in commercial broiler farms across Thailand.

**Materials and Methods:**

Four fecal samples were collected from each of the ten broiler farms in seven provinces of eastern and central Thailand. Oocysts were identified through morphological examination and confirmed through species-specific multiplex polymerase chain reaction. *E. tenella* isolates were propagated and subjected to *in vivo* anticoccidial sensitivity testing (AST) against four drugs: Nicarbazin (NIC), salinomycin (SAL), monensin (MON), and a combination of MON and NIC. Experimental infection trials were conducted on Ross 308 broiler chicks to assess weight gain, fecal oocyst shedding, lesion scores, and anticoccidial index (ACI) values.

**Results:**

*E. tenella* and *Eimeria praecox* were the most prevalent species (40%), followed by *Eimeria acervulina*, *Eimeria brunetti*, and *Eimeria mitis* (20%). Mixed-species infections were detected in 50% of samples. The AST results showed that field isolates were sensitive to NIC, MON, and the MON + NIC combination, with ACI values of 172.51, 175.49, and 174.21, respectively. In contrast, SAL showed an ACI of 158.81, indicating resistance. All treated groups demonstrated reduced oocyst shedding and improved weight gain compared to untreated infected controls, though lesion score differences among treatments were not statistically significant.

**Conclusion:**

This study constitutes the first comprehensive report on anticoccidial drug resistance in *E. tenella* from broiler farms in Thailand. The findings indicate high efficacy of NIC, MON, and their combination, while revealing emerging resistance to SAL. These results provide crucial insights for revising coccidiosis control strategies and support the need for ongoing monitoring and development of alternative therapeutics to mitigate resistance evolution.

## INTRODUCTION

Poultry products, particularly meat and eggs, are vital sources of dietary protein. Among all poultry species, chickens are the most significant globally, accounting for approximately 90% of the total poultry population worldwide [1]. Poultry coccidiosis, caused by the Apicomplexan parasite *Eimeria*, is one of the most economically impactful diseases affecting poultry worldwide [2]. This parasite primarily targets the intestinal epithelium, leading to impaired growth and reduced feed conversion efficiency in poultry [2].

At present, seven *Eimeria* species have been identified in chickens: *Eimeria necatrix*, *Eimeria tenella*, *Eimeria brunetti*, *Eimeria maxima*, *Eimeria acervulina*, *Eimeria mitis*, and *Eimeria praecox*. Chickens across various ages and breeds are susceptible to coccidiosis [3]. *E. tenella* is considered one of the most pathogenic species within the genus *Eimeria*. Infections with *E. tenella* in chickens result in clinical signs, such as lethargy, ruffled feathers, and bloody feces. The principal pathological findings include thickening of the intestinal wall and petechial hemorrhages [4].

Although clinical coccidiosis has been documented globally, with epidemiological studies reported from Brazil [5, 6], Colombia [7], Korea [8], and China [9, 10], no comprehensive survey on anticoccidial resistance has yet been conducted in Thailand. The number of *Eimeria* oocysts per gram (OPG) of litter follows a consistent pattern throughout the flock lifecycle, and these data can be utilized to evaluate the effectiveness of coccidiosis control programs. Furthermore, *Eimeria* species can be identified and distinguished by microscopic examination of species-specific intestinal lesions and oocyst morphology [11]. However, frequent mixed-species infections in field conditions complicate definitive diagnosis. Molecular tools, such as polymerase chain reaction (PCR) can reliably detect *Eimeria* infections in broilers. PCR enhances diagnostic accuracy by minimizing the risk of species misidentification due to overlapping morphometric traits and by detecting low-intensity infections often missed during gross examinations [12].

In Thailand, coccidiosis frequently arises during the later stages of broiler production, a period often characterized by the withdrawal of anticoccidial additives from feed. Effective coccidiosis control requires an integrated strategy involving stringent biosecurity measures combined with the strategic use of anticoccidial drugs. These drugs are broadly classified into ionophores, polyether antibiotics, and synthetic chemicals [13]. Common ionophores include salinomycin (SAL), narasin, monensin (MON), lasalocid, maduramicin, and semduramicin. Synthetic chemicals encompass agents, such as amprolium, toltrazuril, clopidol, and nicarbazin (NIC).

The widespread emergence of drug resistance has compromised the efficacy of these treatments. Therefore, combining ionophores with synthetic anticoccidials is often necessary to disrupt the life cycle of *Eimeria* or eliminate its oocysts [14]. Prolonged drug use may induce genetic modifications in *Eimeria* species, although practical molecular tools for detecting such changes are currently lacking. As a result, anticoccidial sensitivity tests (ASTs) must be conducted *in vivo* to evaluate drug efficacy. These tests assess parameters including lesion scores, oocyst shedding (measured as OPG), body weight gain (BWG), mortality rates, or integrated indices combining these factors [6].

In Thailand, AST implementation remains limited due to insufficient laboratory infrastructure and historically low awareness of resistance issues. In contrast, countries, such as Egypt [15], Iran [16], and India [17] routinely employ ASTs to guide anticoccidial drug selection.

Despite the global recognition of *Eimeria* spp. as a major etiological agent of coccidiosis in poultry, regional variations in species prevalence, infection dynamics, and anticoccidial resistance profiles remain underexplored. Although epidemiological investigations have been conducted in several countries, such as Brazil, Colombia, Korea, and China [5–10], Thailand lacks a systematic and comprehensive survey assessing the prevalence of *Eimeria* species and their resistance to widely used anticoccidial agents. In particular, no field-based studies have yet evaluated the sensitivity of *E. tenella*, the most pathogenic and economically significant species, to commonly administered anticoccidials under Thai broiler production conditions. Furthermore, while molecular tools, such as PCR have been widely adopted elsewhere for accurate species identification, their integration into large-scale field surveillance in Thailand is minimal. The absence of *in vivo* AST data, compounded by inconsistent usage of prophylactic agents and limited infrastructure for resistance monitoring, poses significant challenges to evidence-based control strategies. This knowledge gap restricts the optimization of drug rotation and integrated parasite management programs, increasing the risk of therapeutic failure and economic loss.

This study was designed to bridge the aforementioned gaps by conducting the first large-scale field investigation to estimate the prevalence and anticoccidial drug sensitivity of *E. tenella* isolates from commercial broiler farms in Thailand. Specifically, the study aimed (1) to identify and characterize *Eimeria* species present in fecal samples collected from geographically diverse broiler farms using a combination of microscopic and molecular (PCR-based) techniques, and (2) to evaluate the *in vivo* efficacy of four commonly used anticoccidial drugs – NIC, SAL, MON, and a MON – NIC combination – against *E. tenella* field isolates. The findings are expected to inform regional anticoccidial use policies and support the development of more effective, evidence-based strategies for coccidiosis prevention and control in Thai poultry production systems.

## MATERIALS AND METHODS

### Ethical approval

The study received the approval from the Institutional Animal Care and Use Committee (IACUC) at Rajamangala University of Technology, Tawan-Ok (Permit Number: RMUTTO-ACUC-1-2024-012). The study followed the ARRIVE guidelines for *in vivo* studies.

### Study period and location

The study was conducted from January 2022 to November 2022. We collected fecal samples from ten commercial broiler farms in eastern Thailand. The samples were processed at the Alternative to Antibiotics (ATA) Research Unit, Rajamangala University of Technology Tawan-Ok.

### Sample collection

The sample size was calculated using Epitools (https://epitools.ausvet.com.au/samplesize). Farms were selected regardless of their history of drug use or previous coccidiosis issues to ensure that the isolates accurately represented the broiler industry. None of the farms had a record of vaccination against coccidiosis. Most farms implemented a shuttle program for anticoccidial treatment: starter feed, enriched with a combination of synthetic chemicals and ionophores, was administered during the first 1–10 days of the chicks’ lives, followed by grower feed supplemented with an ionophore agent from days 11 to days 24, after which finisher feed was provided without any anticoccidial treatment.

Samples were collected when birds were between 4 and 7 weeks old, during which no anticoccidial drugs were included in the feed, resulting in a higher incidence of coccidiosis. We aseptically collected fresh fecal samples (30–50 g each) from the four corners and the center of each chicken house. Four samples from each farm were placed in sterile plastic bags containing approximately 300 g of feces and homogenized thoroughly. Subsequently, these samples were trans-ported to the alternative to antibiotics Research Unit at Rajamangala University of Technology, Tawan-Ok.

The fresh fecal droppings were then transferred to a 250-mL container containing 2.5% potassium dichromate solution and aerated for 48 h at 27°C to facilitate the sporulation of oocysts. Oocyst enumera- tion was performed using the McMaster counting technique, with the results expressed as OPG of feces [18, 19]. The OPG values, including the maximum, minimum, and average counts, were calculated, and triplicate observations were conducted for each sample to ensure accuracy. The sporulated oocysts were stored at 4°C until further analysis.

### Identification of Eimeria species

#### Microscopic examination

We identified *Eimeria* species using an Olympus BX43 microscope (Olympus, Japan) with a 40 × objective lens. The identification criteria were oocyst size, shape, presence or absence of a micropyle, and sporulation time [20, 21].

#### DNA extraction and molecular methods

Species confirmation was conducted through a multiplex PCR assay with seven species-specific primer pairs to amplify the ITS1 region of *Eimeria* spp. [22]. The oocysts were washed with distilled water to eliminate potassium dichromate. For genomic DNA extraction, the walls of approximately 10^5^ oocysts were disrupted using a tissue grinder at 4°C to release sporozoites. The samples were centrifuged at 1000 × *g* for 5 min, and the supernatant was transferred to a 1.5-mL microtube containing 2.5 mL of Proteinase K (20 mg/mL), followed by incubation at 50°C for 30 min.

DNA extraction was performed using the E.Z.N.A. Micro Elute Genomic DNA Kit (Omega, USA), according to the manufacturer’s protocol, with minor modifi-cations. Briefly, the samples underwent a pretreatment using a tissue grinder equipped with a probe (Omni, GA, USA) in 400 μL of lysis solution, followed by the subse-quent steps as outlined earlier. The DNA concentration was measured using a NanoDrop 2000 spectrophoto-meter (Thermo Fisher Scientific, Waltham, MA, USA).

Multiplex PCR was conducted in a 25 μL reaction volume consisting of 1 μL of genomic DNA template (56.5 ng/μL), 0.5 μL of each primer (20 μM, synthesized by Bio Basic Co., Ltd., Toronto, Canada), 12.5 μL of 2 × GoTaq® green master mix (Promega, MI, USA), and 10.5 μL of distilled deionized water. DNA was initially denatured at 96°C for 5 min, followed by 30 cycles of amplification at 94°C for 1 min, 65°C for 2 min, and 72°C for 1 min, concluding with a final extension at 72°C for 7 min.

Genomic DNA purified from the live attenuated vaccine strains of each of the five *Eimeria* species (*E. acervulina*, *E. maxima*, *E. tenella*, *E. mitis*, and *E. praecox*; Evant, Hipra, Spain) and two additional *Eimeria* species (*E. necatrix* and *E. brunetti*; Evalon, Hipra, Spain) were utilized as positive controls, while nuclease-free water served as the negative control throughout the multiplex PCR assay. The amplification products were analyzed by gel electrophoresis in 2% (w/v) tris-acetate-ethylenediaminetetraacetic acid buffer at 100 V for 30 min. The gels were stained with 0.01% (v/v) SafeView nucleic acid dye (NBS Biologicals, UK) and examined under ultraviolet light (WiseDoc, Daihan Scientific, Korea).

### Isolation of *E. tenella*

Before *Eimeria* infection, all experimental chicks were raised in wire pens within a coccidia-free facility. They were then transferred to experimental cages located in a distinct area, where the birds were inoculated and maintained until the conclusion of the experimental period.

*E. tenella* oocysts were isolated using the single oocyst separation technique (Method 1), as described by Khalafalla and Daugschies [23]. Briefly, glass slides were sectioned into small cubes, onto which a layer of prewarmed 5% gelatin was applied and allowed to solidify for 10 min at 4°C. Subsequently, a mixture comprising 0.5 mL of prewarmed 5% gelatin and 0.5 mL of oocyst suspension (100 oocysts/mL) was introduced and maintained at 4°C for an additional 10 min. The slides were examined microscopically, and individual sporulated oocysts were excised meticulously. These isolated oocysts were placed into a gel capsule and inoculated into chickens.

On day 7 post-inoculation, the cecal contents were gathered separately and analyzed individually using single-use equipment and autoclaved materials to reduce the risk of contamination. Feces were collected in trays containing a 2.5% (w/v) potassium dichromate solution. After washing, the oocysts were sporulated through forced aeration. *Eimeria* species were identified based on the location and appearance of gross lesions in the intestine, along with microscopic examination and measurement of the oocysts [24, 25]. This was followed by verification of a single species of the oocyst using multiplex PCR.

The *E. tenella* field isolate was passaged through coccidia-free broilers for propagation and pathogenicity analysis. To assess the pathogenicity and dosage of *Eimeria* before the AST, a cohort of 25 coccidia-free day-old broiler chickens was reared until they reached 14 days of age. Chickens of similar weight were randomly allocated into five groups of five each. Groups 1, 2, 3, and 4 were inoculated with *E. tenella* using four doses of oocysts: 2,500, 5,000, 10,000, and 20,000, respectively. A control group was included for comparative purposes.

Fecal samples collected 6 and 9 days post-inoculation were incubated with shaking for 5 days and then examined microscopically to count the quantity of sporulated oocysts. Ten days after inoculation, the birds were individually weighed and euthanized for necropsy and lesion scoring. The inoculation dose was adjusted to achieve a lesion score of 2–3 on the Johnson and Reid scale [26] while preventing mortality.

### AST

A total of 144 1-day-old Ross 308 male broiler chickens were randomly divided into six groups with equal average weights, with each group including four replicates of six birds. The diet composition was suitable for the growth stage of the birds (Table 1) and met the NRC [27] requirements. The birds were provided filtered water and feed ad libitum.

**Table 1:** Ingredients and nutrient contents of basal diets during different experimental periods.

Ingredients (%)	Starter (days 0–10)	Grower (days 11–21)
Corn	52.76	58.17
Soybean meal (48% CP)	34.93	31.55
Rice solvent bran	5.92	4.86
Palm oil	2.50	2.50
Monodicalciumphosphate 21%	1.11	0.67
Calcium carbonate	1.18	0.86
L-lysine	0.28	0.21
DL-methionine	0.38	0.33
L-threonine	0.11	0.06
L-valine	0.07	0.04
Salt	0.30	0.30
Phytase + NSP end 100 g/t (−70 kcal/kg)	0.01	0.01
Choline chloride 60%	0.28	0.27
Premi×^1^	0.18	0.18
Total	100.00	100.00
Nutrient contents		
Metabolizable energy (kcal/kg)	2,975.00	3,050.00
CP (%)	23.00	21.50
Fat (%)	4.76	4.91
Crude fiber (%)	4.12	3.93
Calcium (%)	0.95	0.75
Total phosphorus (%)	0.88	0.76
Available phosphorus (%)	0.50	0.42
Salt (%)	0.34	0.33
Choline, mg/kg	1,700.00	1,600.00
Digestible lysine (%)	1.32	1.18
Digestible methionine (%)	0.68	0.62
Digestible methionine+cystine (%)	1.00	0.92
Digestible threonine (%)	0.88	0.79
Digestible tryptophan content (%)	0.26	0.24
Digestible valine (%)	1.00	0.91

1 Provides each kg of diet: Vit. A: 12000 IU, Vit. D3: 5000 IU, Vit. E: 65.0 mg, Vit. K3: 3.0 mg, Vit. B1: 3.0 mg, Vit. B2: 6.5 mg, Vit. B6: 3.2 mg, Vit. B12: 0.02 mg, Nicotinic acid: 16.0 mg, Folic acid: 1.8 mg, Biotin: 180.0 mg, D-calcium: 16 mg, Copper: 80.0 mg, Iodine: 5.0 mg, Selenium: 100.0 mg, Iron: 40.0 mg, Manganese: 80.0 mg, Zinc: 60.0 mg, Cobalt: 100.0 mg. CP=Crude protein, d=Days of age

Experimental rations were prepared by mixing feed ingredients with commercial anticoccidial drug premixes containing the required concentrations of anticoccidials. These ingredients were added to the starter and grower diets at specified levels in each study. All unconsumed feed was weighed and discarded on a weekly basis.

Chickens were housed in heat-treated, wire-floored battery pens (2 m² surface area) within a coccidia-free facility. The temperature was maintained between 29°C and 32°C by electric heating using 100-W bulbs. Red light was continuously provided for 24 h during the experimental period.

The groups were organized as follows:

1. Non-infected non-medicated control group (NNC), which received the basal diet and was not inoculated with *Eimeria*;
2. Infected non-medicated control group (INC), which received the basal diet and was inoculated with *Eimeria*;
3. The INC group was supplemented with 40 parts-per-million (ppm) NIC per ton of diet (NIC);
4. The INC group was supplemented with 70 ppm SAL per ton of diet (SAL);
5. The INC group was supplemented with 100 ppm MON per ton of diet (MON);
6. The INC group was supplemented with 40 ppm MON plus 40 ppm NIC per ton of diet (MON + NIC) (Table 2).

**Table 2:** Experimental design outlining the treatment groups, prophylactic anticoccidial agents, dosages, and challenge status used in the AST.

Treatment^1^	Coccidiostat	Dose (ppm)	Eimeria challenge
1 (NNC)	No	-	No
2 (INC)	No	-	Yes
3	NIC	40	Yes
4	SAL	60	Yes
5	MON	100	Yes
6	MON + NIC	40 + 40	Yes

1 Experimental group of four replicates with six individuals each. NNC=Non-infected non-medicated control, INC=Infected non-medicated control, NIC=Nicarbazin, SAL=Salinomycin, MON=Monensin, AST=Anticoccidial sensitivity test, ppm=Parts-per-million

At 14 days, all chickens were weighed and tagged for identification. The mean body weight was 540 g, and no significant differences were observed among the initial weight groups. Subsequently, the chickens were orally infected with 1.5 × 10^4^ sporulated oocysts of *E. tenella* in a 1 mL suspension delivered into the crop. The unmedicated control group remained uninfected and was administered distilled water.

The birds were observed daily, and any deceased birds were collected for necropsy and weight recording. Feces were collected from each post-infection group 6 days post-infection, with one drop collected each day to assess the relative number of OPG of feces. At 21 days of age (7 days post-infection), all chickens were weighed individually (final BW) and euthanized for necropsy.

The chickens’ survival rate, BWG, and oocyst values were recorded. The coccidial lesions observed in the chickens were scored on a scale of 0 to 4 according to the methods described by Johnson and Reid [26]. The ACI was then calculated to evaluate the drug’s effectiveness.

The ACI was calculated using the following formula:

ACI = (Relative BWG + Survival rate) ÷ (Lesion score + Oocyst value)

The oocyst value was calculated as follows:

(Fecal oocyst count of the treated-infected group)/(Fecal oocyst count of the positive control group) × 100%

An ACI value of ≥160 indicates sensitivity, whereas a value <160 signifies resistance [28].

### Statistical analysis

Data were analyzed using GraphPad Prism 8 (GraphPad Software, San Diego, CA, USA). We analyzed body weight, feed intake, lesion scores, and oocyst counts per gram of feces using a one-way analysis of variance followed by a *post hoc* analysis with Tukey’s multiple comparison test to identify statistically significant differences. A difference was considered significant if p < 0.05.

## RESULTS

### Dominance of *Eimeria* species in chickens

Molecular identification revealed that *E. tenella* and *E. praecox* were the predominant species, each detected in 40% of the sampled locations (4/10 farms). This was followed by *E. acervulina*, *E. brunetti*, and *E. mitis*, which were each identified in 20% of locations (2/10 farms). Mixed-species infections involving two or more *Eimeria* species were common, with the most frequently observed co-infection being *E. tenella* and *E. brunetti* (Table 3).

**Table 3:** Species-specific distribution of *Eimeria* oocysts identified through microscopic examination and PCR amplification.

Sample	Type of oocyst observed during the initial microscopic examination	Identification by PCR amplification
NR1	*E. tenella, E. necatrix,* and *E. praecox*	*E. tenella, E. necatrix,* and *E. acervulina*
NR2	*E. acervulina, E. necatrix*	*E. acervulina*
SB1	*E. praecox, E. necatrix,* and *E. brunetti*	*E. praecox, E. brunetti*
PB1	*E. tenella, E. necatrix,* and *E. maxima*	*E. tenella, E. brunetti*
SK1	*E. necatrix, E. praecox*	*E. praecox*
SK2	*E. tenella, E. necatrix*	*E. tenella*
CS1	*E. necatrix, E. praecox*	*E. praecox*
CB1	*E. tenella, E. necatrix,* and *E. mitis*	*E. tenella, E. mitis*
CB2	*E. maxima, E. brunetti*	*E. maxima, E. praecox*
RY1	*E. necatrix, E. mitis, E. praecox*	*E. mitis*

PCR=Polymerase chain reaction, *E. tenella=E. tenella, E. necatrix=* *E. necatrix, E. praecox=E. praecox, E. acervulina=E. acervulina, E. brunette=E. brunetti, E. maxima=E. maxima, E. mitis=E. mitis*, NR1=Nakhon Ratchasima1, SB1=Saraburi1, PB1=Prachinburi1, SK1=Sakaeo1, CS1=Chachoengsao1, CB1=Chonburi1, RY1=Rayong1

### Relationship between geographical location, OPG, and coccidiostat usage

Of the ten farms surveyed, six farmers responded to the questionnaire concerning the use of in-feed coccidiostats for coccidiosis prevention (Table 4). The average oocyst per gram (OPG) count among fecal samples was 3.713 ± 2.915. Two farms in Sakaeo and Chachoengsao Provinces used SAL as a prophylactic anticoccidial. In these farms, OPG levels ranged from 3.8 to 7.9 × 10^3^. The remaining four farms, located in Saraburi, Sakaeo, Chonburi, and Rayong, did not use any anticoccidial feed additives. Their OPG levels ranged from 0.2 to 4.1 × 10^3^.

**Table 4:** Relationship between geographical location, OPG feces, and currently used drugs.

Sample	Location	Age of the flock (days)	Average number of OPG of positive samples (min.–max.)	In-feed coccidiostat
NR1	Nakhon Ratchasima	21	2,879 ± 2,225 (0–27,600)	Nd
NR2	Nakhon Ratchasima	21	666 ± 342 (0–18,933)	Nd
SB1	Saraburi	41	4,153 ± 3,338 (300–6,160)	No
PB1	Prachinburi	28	8,267 ± 6,372 (100–75,533)	Nd
SK1	Sakaeo	31	7,983 ± 2,970 (4,300–11,700)	Salinomycin
SK2	Sakaeo	45	230 ± 98 (0–300)	No
CS1	Chachoengsao	23	3,849 ± 3,662 (0–13,200)	Salinomycin
CB1	Chonburi	28	5,808 ± 4,022 (300–91,700)	Nd
CB2	Chonburi	35	2,728 ± 6,686 (0–23,200)	No
RY1	Rayong	42	573 ± 845 (0–2,600)	No

nd=Not completed, OPG=Oocyst per gram, NR1=Nakhon Ratchasima1, SB1=Saraburi1, PB1=Prachinburi1, SK1=Sakaeo1, CS1=Chachoengsao1, CB1=Chonburi1, RY1=Rayong1

### AST results

Feed consumption was highest in the MON-treated group (MON) and lowest in the INC group. The NNC group had the lowest feed conversion ratio (FCR). BWG was significantly greater in both the NNC and all medicated infected groups compared to the INC group. Notably, chickens treated with NIC and the MON-NIC combination showed significant improvements, with differences in weight gain observed between these two treatments (Table 5).

**Table 5:** Summary of performance results of experimental birds between 14 and 21 days old.

Treatment	Initial body weight^1^ (g)	Final body weight^1^ (g)	BWG^1^ (g)	Total consumed feed (g)	Average feed conversion ratio
1, NNC	578.29 ± 44.22^a^	1,159.79 ± 63.83^a^	581.50 ± 46.10^ac^	44,150	3.16
2, INC	566.45 ± 60.08^a^	940.66 ± 133.09^b^	414.70 ± 84.10^b^	42,020	4.22
3, NIC	574.04 ± 47.33^a^	1,138.08 ± 89.15^a^	564.04 ± 53.17^a^	43,000	3.17
4, SAL	571.87 ± 45.89^a^	1,132.41 ± 64.37^a^	560.54 ± 30.91^ac^	42,950	3.19
5, MON	609.87 ± 42.55^a^	1,170.45 ± 112.24^a^	560.58 ± 77.18^ac^	44,700	3.32
6, MON + NIC	587.37 ± 57.38^a^	1,109.28 ± 138.38^a^	521.83 ± 70.80^c^	44,540	3.55

1 The values (n = 24) are represented as the mean ± standard deviation, ^a-c^Among columns; Numbers with different superscripts differ significantly (p < 0.05) for a given parameter. NNC=Non-infected non-medicated control, INC=Infected non-medicated control, NIC=Nicarbazin, SAL=Salinomycin, MON = Monensin, BWG=Body weight gain

No oocysts were detected in the NNC group. Among the medicated groups, the MON + NIC group had the lowest mean oocyst count (2.71 × 10^4^ ± 1.17 per gram of feces), whereas the SAL group exhibited the highest count (4.38 × 10^4^ ± 2.21 per gram). All medicated groups showed significantly reduced fecal oocyst counts compared to the INC group (p < 0.05).

Lesion scoring revealed that cecal lesions were absent in the NNC group but present in all infected groups. The INC group had the highest lesion scores. Among treated groups, the MON + NIC combination yielded the lowest lesion scores, suggesting the highest therapeutic efficacy, whereas the SAL group had the highest scores among treated groups (Table 6). However, differences in lesion scores among the medicated groups were not statistically significant.

**Table 6:** Results of OPG and LS.

Treatment	Average OPG × 10^4^	Average LS
1, NNC	0^a^	0^a^
2, INC	20.07 ± 15.21^b^	3.37 ± 1.09^b^
3, NIC	4.38 ± 1.88^c^	2.25 ± 1.15^b^
4, SAL	7.02 ± 2.21^c^	2.58 ± 1.05^b^
5, MON	3.73 ± 1.51^c^	2.29 ± 1.26^b^
6, MON + NIC	2.71 ± 1.17^c^	2.00 ± 1.21^b^

^1^The values (n = 24) are represented as the mean ± standard deviation, ^a-c^Among columns; Numbers with different superscripts differ significantly (p < 0.05) for a given parameter. OPG=Oocyst per gram, LS=Lesion score, NNC=Non-infected non-medicated control, INC=Infected non-medicated control, NIC=Nicarbazin, SAL=Salinomycin, MON=Monensin

The anticoccidial index (ACI) results indicated sensitivity to NIC (ACI = 172.51), MON (ACI = 175.49), and the MON + NIC combination (ACI = 174.21), while resistance was observed for SAL (ACI = 158.81) (Table 7).

**Table 7:** ACI and sensitivity classification of each drug evaluated in this study.

Treatment	BWG rate (%)	Survival rate (%)	Oocyst value	ACI	Sensitivity
1, NNC	100	100	0	200	-
2, INC	71.31	100	100	67.93	-
3, NIC	96.61	100	21.85	172.51	Sensitive
4, SAL	96.39	100	35	158.81	Resistance
5, MON	96.38	100	18.60	175.49	Sensitive
6, MON + NIC	89.73	100	13.52	174.21	Sensitive

NNC=Non-infected non-medicated control, INC=Infected non-medicated control, NIC=Nicarbazin, SAL=Salinomycin, MON=Monensin, ACI=Anticoccidial index, BWG=Body weight gain

## DISCUSSION

This study aimed to estimate the prevalence of *Eimeria* species and evaluate the anticoccidial drug sensitivity of *E. tenella* field isolates in commercial broiler farms across Thailand.

### Economic impact of coccidiosis in Thailand

Chicken coccidiosis caused by *Eimeria* species is a major contributor to economic losses in the global poultry industry. Although no official economic estimates exist for Thailand, preliminary observations from farms experiencing coccidiosis outbreaks showed an increase in mortality rates by 0.9% and a rise in FCR by 0.1. While these values reflect production losses, they likely represent only a fraction of the overall burden compared to countries, such as India [29] and Romania [30], where treatment costs are also considered.

### Epidemiological insights and environmental factors

Regular epidemiological monitoring is essential for designing effective prevention and control strategies. The average OPG value observed in this study (3.7 × 10³) was similar to values reported in China [31] and lower than those reported in Colombia (1.9 × 10^4^) [7]. Although OPG values are useful for monitoring infection progression, this study only included a single sampling per farm, which may have missed peak shedding periods.

Our results are consistent with findings from Bangladesh, where higher coccidiosis incidence was reported in tropical regions with high humidity and temperature, conditions that favor oocyst sporulation and transmission [32]. In addition, variations in hygiene and farm management practices likely contributed to differences in *Eimeria* prevalence across farms [33].

### Species distribution and comparative prevalence

In Thailand, *E. tenella* and *E. praecox* were the most prevalent species (40%), followed by *E. acervulina*, *E. brunetti*, and *E. mitis* (20%), and *E. necatrix* and *E. maxima* (10%). These findings are consistent with a study from Nigeria that reported high prevalence rates for *E. tenella* (77%) and *E. acervulina* (44%) [34], but differ from reports in Korea, where *E. acervulina* was found in 98.6% of cases [8].

The dominance of *E. tenella* in Thai broiler farms may be due to its aggressive reproductive potential and pathogenicity. This is further supported by previous literature documenting the strain’s widespread impact in poultry [35, 36]. The frequent use of certain vaccines may also select for resilient strains like *E. praecox*, contributing to its observed prevalence. Species distribution is influenced by oocyst load, host immunity, and the biological characteristics of individual *Eimeria* species [37].

### Mixed infections and environmental contributors

Our study found that 50% of the farms harbored mixed-species infections, consistent with data from Argentina, where co-infections reached 81.08% [38]. Several environmental and operational factors may explain such variability across regions. Warmer and humid climates enhance sporulation and transmission, while farm-level variables, such as litter management and biosecurity practices play critical roles in parasite survival and dissemination [39, 40].

While our study included farms from densely populated poultry-producing regions in central and eastern Thailand, the relatively small sample size and limited geographic scope may restrict the generaliz-ability of the findings. Future studies incorporating broader geographic sampling are recommended to validate and extend these observations.

### Drug resistance and anticoccidial efficacy

Although anticoccidial drug resistance has been extensively studied in other regions by Tan *et al*. [41] and Vereecken *et al*. [42], limited information is available from Thailand. In this study, the sensitivity of *E. tenella* to four widely used anticoccidials – NIC, SAL, MON, and MON + NIC was assessed. Parameters measured included BWG, lesion scores, oocyst output, and mortality.

All infected chickens showed reduced BWG, a hallmark of clinical coccidiosis. Birds treated with NIC and MON + NIC showed significantly greater weight gains than untreated controls. Furthermore, uninfected groups outperformed all infected groups in terms of growth and feed efficiency, underscoring the production impact of even subclinical infections. These findings support previous research showing that coccidiosis impairs productivity despite prophylactic drug use [6].

### Emergence of drug resistance

Though treatments are considered effective when they preserve weight gain and reduce lesions, repeated use of the same drugs may promote resistance. Continued reliance on NIC, MON, and their combination raises concerns about emerging multidrug resistance in Thai poultry farms [43]. Resistance has already been documented for MON [44], NIC [45], and their combination [46].

All four medicated groups showed significantly reduced oocyst counts compared to controls, aligning with findings from China, where high oocyst excretion persisted despite treatment [41]. However, reductions in lesion scores were less consistent across groups. Relying on a single outcome metric for drug efficacy may be misleading [47]; hence, the ACI was employed as a composite measure.

### Drug sensitivity patterns in field isolates

Field isolates of *E. tenella* demonstrated sensitivity to most of the tested drugs, with the exception of SAL. Resistance to SAL was reflected in the ACI and is consistent with earlier reports documenting varying resistance levels to this ionophore [48, 49]. Ionophores and chemical anticoccidials differ in their mode of action – ionophores disrupt ion transport, while chemicals interfere with metabolic pathways [31]. Resistance development in ionophores has been linked to alterations in parasite membrane biochemistry [50].

This study provides the first comprehensive field-based assessment of anticoccidial sensitivity in *E. tenella* isolates from Thailand. It also represents the inaugural report on the molecular prevalence of *Eimeria* spp. in Thai commercial broiler farms. These findings offer critical insights for guiding future control programs and underline the importance of resistance surveillance in regions with intensive poultry production.

## CONCLUSION

This study provides the first field-based molecular and phenotypic characterization of *Eimeria* spp., particularly *E. tenella*, in commercial broiler farms across Thailand. Molecular diagnostics identified *E. tenella* and *E. praecox* as the most prevalent species (40%), with *E. acervulina*, *E. brunetti*, and *E. mitis* also detected. Mixed infections were frequent (50%), reflecting complex epidemiological dynamics under tropical field conditions. AST revealed that field isolates of *E. tenella* remained sensitive to NIC, MON, and their combination (ACI > 160), whereas reduced efficacy and potential resistance were observed with SAL (ACI = 158.81).

These findings carry significant practical implications for disease management strategies in Thai poultry operations. The high prevalence of pathogenic *Eimeria* spp. and evidence of emerging drug resistance highlight the urgent need to revise coccidiosis control programs. The continued effectiveness of NIC and MON supports their cautious inclusion in rotation programs, while the resistance to SAL warrants reconsideration of its routine use.

A major strength of this study is the integrated approach combining molecular diagnostics, *in vivo* sensitivity assays, and field-level epidemiological data, which enhances the reliability of conclusions regarding species distribution and drug response. This study also establishes a much-needed baseline for anticoccidial resistance monitoring in Thailand.

However, certain limitations must be acknowledged. The relatively small number of farms (n = 10), all from central and eastern Thailand, may limit the broader generalizability of the findings. Single-point fecal sampling may have missed peak oocyst shedding periods, potentially underestimating parasite burden.

Future research should expand to a larger and more geographically diverse sample set, incorporate longitudinal sampling, and integrate molecular tools to monitor genetic markers of resistance. In addition, investigating the efficacy of alternative control measures, such as live vaccines or phytogenic additives, could complement chemical-based strategies.

This study highlights the widespread occurrence and complex nature of *Eimeria* infections in Thai broiler farms, providing compelling evidence for the evolving resistance landscape. These insights are essential for guiding evidence-based anticoccidial usage and developing sustainable parasite control frameworks to safeguard poultry health and productivity in Thailand.

## DATA AVAILABILITY

All the generated data are included in the manuscript.

## AUTHORS’ CONTRIBUTIONS

ST and NC: Conceived and designed the study and data analyses. ST, SB, RA, and NC: Conducted the study and drafted and revised the manuscript. All authors have read, reviewed, and approved the final version of the manuscript.
